# Supplementary material for: Consumers discard a lot more food than widely believed: Estimates of global food waste using an energy gap approach and affluence elasticity of food waste
Source: PLoS One. 2020 Feb 12;15(2):e0228369. doi: 10.1371/journal.pone.0228369 (PMC7015318; doi:10.1371/journal.pone.0228369)
Supplement: S2 File — (DOCX) [file pone.0228369.s002.docx]

Deriving Equation (1) in manuscript

On the basis of methodology used by Hall et al. 2009, we can write the following two equations:

$FW=Food Availability \left( FA \right)-Food Intake (FC)$ (S1)

$FC=\Delta Bodyweight \left( BW \right).\rho+Energy Expenditure (E)$ (S2)

As presented by equation (S1), FW is calculated as the difference between food available for consumption (in kcal/cap/day) and actual food intake/consumption (in kcal/cap/day).

Equation (S2) is the energy balance equation from Hall et al (2009). Energy intake from food, equals the energy expenditure(E) and the rate of change in stored body energy (ΔBWρ). BW presents the body weight in kg, ρ the energy density of the change in body weight in kcal/kg and E the energy expenditure in kcal/cap/day, respectively. Since the unit ΔBWρ should also be in kcal/cap/day and the daily change in BW cannot be substantial: Hall et al. 2009 estimated it to be less than 10 kcal/day in 30 years of US data, we assume the daily change in BW to be zero. Also for an optimally healthy population, food consumption is equal to the energy requirement (WHO 2015) and in absence of health characteristics modelling in our final analysis we assume the given state to be optimal. Moreover, as we do not have a time series information on evolution of BW in most countries, equation (S2) is simplified to the following:

$FC=E$ (S2’)

E is estimated by the (per capita) Energy Requirement (ER) from the Human Energy Requirements Report by the FAO, World Health Organisation (WHO) and United Nations University (UNU)(2004), by using the following equation:

$E=PAL*BMR$ (S3)

Where PAL is the Physical Activity Level and BMR is the Basal Metabolic Rate in kcal/cap/day. The BMR value is used to account for differences in body size, composition, age and gender; the PAL value accounts for different lifestyles (FAO 2001).

BMR is calculated for each country by using an equation based on BW, as follows:

${BMR}_{c}=\alpha_{c}{BW}_{c}+C_{c}$ (S4)

The coefficients in (S4) are country-specific, as denoted by the subscript c; they differ by gender and age category (FAO 2001). The country-specific coefficients are determined as the weighted average of the age- and gender-specific coefficients reported by the FAO 2001. Weights are calculated based on a country’s gender and population shares by age category.

$\alpha_{c}=\left[ {population share}_{c}^{FEMALE,15-19}*\alpha_{c}^{FEMALE,15-19}+{population share}_{c}^{FEMALE,20-29}*\alpha_{c}^{FEMALE,20-29}+{population share}_{c}^{FEMALE,30-59}*\alpha_{c}^{FEMALE,30-59}+{population share}_{c}^{FEMALE,\geq60}*\alpha_{c}^{FEMALE,\geq60} \right]+\left[ {population share}_{c}^{MALE,15-19}*\alpha_{c}^{MALE,15-19}+{population share}_{c}^{MALE,20-29}*\alpha_{c}^{MALE,20-29}+{population share}_{c}^{MALE,30-59}*\alpha_{c}^{MALE,30-59}+{population share}_{c}^{MALE,\geq60}*\alpha_{c}^{MALE,\geq60} \right]$ (S5)

The age-specific population shares are derived from the World Bank (2016b).

Substituting (S3), (S4) and (S5) in (S2’), we get (S6)

$FI=PAL*\left[ \left\{ \sum_{a,g} {population share}_{c}^{g,a}*\alpha_{c}^{g,a} \right\}{BW}_{c}+\left\{ \sum_{a,g} {population share}_{c}^{g,a}*C_{c}^{g,a} \right\} \right]$ (S6)

Finally substituting (S6) in (S1) gives us equation (1) in the article.
