# Supplementary material for: Consumers discard a lot more food than widely believed: Estimates of global food waste using an energy gap approach and affluence elasticity of food waste
Source: PLoS One. 2020 Feb 12;15(2):e0228369. doi: 10.1371/journal.pone.0228369 (PMC7015318; doi:10.1371/journal.pone.0228369)
Supplement: Attachment — Submitted filename: Response to Reviewers.pdf [file pone.0228369.s004.pdf]

Dear PLOS One Editor(s),

Thank you for giving us the chance to respond and revise this manuscript. We receive the feedback from out reviewers with much appreciation.

We have very diligently, and to best of our ability, responded to all the comments raised by the reviewers. We used the document with all the comments, provided to us by the journal editor and added our responses in the same document. As there were many comments, we highlighted our response with grey background, for ease of trackability and reading.

Each response follows the comment it is meant to address. We have also made extensive changes to text and figures in order to incorporate these comments and suggestions from reviewers. Oftentimes but not always part of changed/added text is reproduced in the response to comments. A few comments however are merely addressed here and do not lead to a change in the manuscript.

We hope we have managed to address all the concerns raised in comments.

Sincerely,

Monika

Enclosed

Response to Journal requirements

Response to Reviewer 1

Response to Reviewer 2

Response to Reviewer 3

Journal requirements

When submitting your revision, we need you to address these additional requirements.

1. Please ensure that your manuscript meets PLOS ONE's **style requirements**, including those for file naming. The PLOS ONE style **templates** can be found at

http://www.journals.plos.org/plosone/s/file?id=wjVg/PLOSOne_formatting_sample_main_body.pdf and http://www.journals.plos.org/plosone/s/file?id=ba62/PLOSOne_formatting_sample_title_authors_affiliations.pdf

Response: We did follow these templates with initial submission as well, and seem to have missed a few details which we tried to accommodate (as seen in tacked changed manuscript).

2. We note that **Figure 1** in your submission contain copyrighted images.

All PLOS content is published under the Creative Commons Attribution License (CC BY 4.0), which means that the manuscript, images, and Supporting Information files will be freely available online, and any third party is permitted to access, download, copy, distribute, and use these materials in any way, even commercially, with proper attribution. For more information, see our copyright guidelines: http://journals.plos.org/plosone/s/licenses-and-copyright.

We require you to either (a) present written permission from the copyright holder to publish these figures specifically under the CC BY 4.0 license, or (b) remove the figures from your submission:

a. You may seek permission from the original copyright holder of Figure(s) [#] to publish the content specifically under the CC BY 4.0 license.

We recommend that you contact the original copyright holder with the Content Permission Form (http://journals.plos.org/plosone/s/file?id=7c09/content-permission-form.pdf) and the following text:

“I request permission for the open-access journal PLOS ONE to publish XXX under the Creative Commons Attribution License (CCAL) CC BY 4.0 (http://creativecommons.org/licenses/by/4.0/). Please be aware that this license allows unrestricted use and distribution, even commercially, by third parties. Please reply and provide explicit written permission to publish XXX under a CC BY license and complete the attached form.”

Please upload the completed Content Permission Form or other proof of granted permissions as an "Other" file with your submission.

In the figure caption of the copyrighted figure, please include the following text: “Reprinted from [ref] under a CC BY license, with permission from [name of publisher], original copyright [original copyright year].”

b. If you are unable to obtain permission from the original copyright holder to publish these figures under the CC BY 4.0 license or if the copyright holder’s requirements are incompatible with the CC BY 4.0 license, please either i) remove the figure or ii) supply a replacement figure that complies with the CC BY 4.0 license. Please check copyright information on all replacement figures and update the figure caption with source information. If applicable, please specify in the figure caption text when a figure is similar but not identical to the original image and is therefore for illustrative purposes only.

Response: Figure 1 has now been modified. Some images have been replaced by those found on the creative commons website image searches, while those where a good substitute could not be found, have been removed and replaced with text alone.

3. Please amend either the title on the online submission form (via Edit Submission) or the title in the manuscript so that they are identical.

Response: The title has been modified in the Online Submission form to match that in the manuscript. The title should now read consistently across the two as – “Consumers discard a lot more food than widely believed: Estimates of global food waste using energy gap approach and affluence elasticity of food waste”

Reviewer 1

Reviewer #1: Review for: ‘‘Consumers discard a lot more food than widely believed: Estimates of global food waste using energy gap approach and affluence elasticity of food waste’’

Summary

Summary:
This paper proposes an indirect method for computing internationally comparable consumer food waste. The method computes food waste on food availability, energy gap and consumer affluence. They also establish a relationship consumer affluence and food waste as well as a threshold where this relationship changes. Overall, this is an interesting exercise and piece of contribution to improve our understanding of the quantity of food wasted world-wide.

I have the following major and minor comments:

Thank you for your time to reflect on this manuscript and share your thoughts with us. As there were many comments, we highlighted our response with grey background, for ease of trackability and reading. Each response follows the comment it is meant to address. We have also made extensive changes to text and figures in order to incorporate these comments and suggestions. Oftentimes part of changed/added text is reproduced in the response to comments below.

Major comments

Major comments:

1. The paper lacks clarity and details on how they are computing some of the estimations. For instance, the authors argue that the relationship between food waste and consumer affluence changes after some threshold. But I was expecting a graph showing this non-linear relationship.

Response: This non-linear relationship has been portrayed in Figure 2a. The plot shows per capita per day calorie waste on the y-axis and per capita Actual Individual consumption expenditure (consumer affluence) on x-axis. The residual plot of this non-linear curve, support this functional form. The threshold level of income in this graph is the point of intersection of the curve with x-axis – after this level of income after which food waste rises rapidly at first and gradually thereafter (as shown by slope of the curve).

The same curve (though restricted to positive observations) is reproduced with additional information on affluence elasticities in Figure 4. Figure 4 in particular, shows a jump in the elasticity numbers (blue scatter points) from zero to very high positive number around the threshold level of income. Beyond this point (as seen from figure 4) the blue curve falls rapidly from a very high values to lower ones and becomes flatter.

But you are right, we did not explicitly include the threshold in the figure. We have now introduced a vertical dashed line at the threshold level will help bring out this information more clearly. Also description has been added to text associated with Figure 4.

"**The figure also shows a vertical dashed line as the threshold level of AIC/capita beyond which consumer food waste turns positive (shown as change from hollow to solid coloured data points in the per capita food waste series) and elasticity jumps from non-existent (zero) to very high levels**."

2. The authors claims estimating affluence elasticity of food waste, an idea I liked. But I am not sure if this is executed correctly because quantifying elasticity involves estimating log-log relationships. But the authors are not exactly estimating such relationships.

Response: we agree that estimating a log-log relationship is the most intuitive way to obtain elasticities. However given that our data supports not a log-log but a linear-log (dependent variable without log and independent variable in logs), we use the following modified version to estimate elasticity.

Function suggested by data is

D = c + b log(A), where D is waste, c is constant, b is slope and A is affluence, which gives us

$$\frac{\partial D}{\partial \log(A)} = b \qquad (1)$$

Elasticity is measured (as you pointed out) by $\frac{\partial \log(D)}{\partial \log(A)}$

With some manipulation we can rewrite the elasticity formula above as follows

$$\frac{\partial \log(D)}{\partial \log(A)} = \frac{\frac{\partial D}{D}}{\partial \log(A)} \qquad (2)$$

Further combining, (1) and (2), we get and expression for elasticity as

$$\boxed{\frac{\partial D}{D.\ \partial \log(A)} = \frac{b}{D}}$$

This boxed expression is the one used to calculate and report elasticity. The elasticity varies across regions due to the differential denominator (D) across regions.

In short, you are right that we are not estimating a log-log function, but we are not claiming the slope estimates to be elasticity either (they would be if it were to be a log-log function as you pointed out). We can calculate elasticity from a not log-log function, with some algebraic manipulation (as above).

3. I would add a bit more discussion on the actual estimation of relationships. I was expecting more details on the estimation and tables.

Response: Thanks for pointing this out, the text now includes an interpretation of the coefficient estimates provided in Table 2. If you could please advise as to what more can be provided to help the manuscript further, we would be happy to oblige. As for the more details on estimation, we provide the script and data to those interested in reproducing our results.

<u>Added/modified text:</u> "**The negative estimates for constant in the relationship shows that FW is not a problem at low levels of affluence, infact, A benefit of using this approach is that using the AIC/cap estimates from Table 2 one can also help identify the income threshold (2450 international 2005 USD) beyond which consumer FW in a country turns positive and starts to increase rapidly. The positive slope estimates tell us that for a 1 percent increase in affluence, FW increases by about 5-6 (576.7/100) calories. Note that while overtime changes in body weight, don't explicitly feature in the regression and in obtaining a single country data, the sample variation in bodyweight across countries (Table 1) allows for this possibility.**"

4. Data related issues: I am wondering if the authors can use a combined ICP data for 2005 and 2011, so that they may also estimate panel regressions.

Response: Great that you brought this up! In-fact at earlier stages of this work, we did entertain the possibility of including a longitudinal component in the estimates. Unfortunately, while it was possible to obtain data on the independent variables (ICP), no such data could be found for the dependent variable (Bodyweight and by implication Food waste) beyond a single year. Happy to hear your thoughts on it further, because it would certainly be an improvement over the current approach.

5. Data related issues: why should we trust the FAOSTAT for food availability but not for food waste or food loss? This requires a bit of justification.

Response: We **<u>don't justify</u>** using the FAO food availability data, as a matter of fact we start by acknowledging the problems that using FAO FBS data entails – "**Apart from being questionable for reasons identified in** (Hall et al. 2009; Svedberg 1999; Hawkesworth Sophie et al. 2010; FAO 2001)**,…**"; and we also explicitly state that – "**While we address the fixed factors issue with FAO approach, we are as guilty as FAO in using the FAO FBS data as a measure of food availability**". But we see your objection and the text now includes, a statement as to why we still do it.

<u>Added text</u>: "**We understand that FBS data is not free of problems and particularly so for lower income countries where subsistence farming is still prevalent [2–5]. Correcting for both supply and demand side problems in measuring FW is however is beyond the scope here. At present only a single step towards correcting the demand side is undertaken in the current work.**"

Minor comments

Minor comments:

There seems some errors and typos in few places (e.g., page 8)
Response: With no specific instances pointed to, we made a few changes to make text on page 8 read better.

Reviewer 2

Reviewer #2: General Comments

We thank you for your very extensive and detailed comments and feed-back, and we found them helpful. It took quite some time and effort, and as you'll see we have, to our best ability and sincerely addressed most of them. As there were many comments, we highlighted our response with grey background, for ease of trackability and reading. We have also made extensive changes to text and figures in order to incorporate these comments and suggestions. Oftentimes part of changed/added text is reproduced in the response to comments below.

General comments

This is an interesting study that investigates relation between food waste and consumer affluence. Mainly, the authors estimated food waste as the differences between food availability and food consumption. Food consumption was calculated based on physical activity level and body weight as few other studies also did. Afterwards, the manuscript presents the fitting of the per capita food waste and income data. Although the study presents interesting finding showing that food waste currently increase with income, the manuscript cannot be considered for publication in the current form due to following reason.

First, the method use for estimating food waste has been reported by previous studies which the authors do not acknowledge in the manuscript. Instead, the authors claim that the applied method is a sophisticated one although it does not account for food requirement of children and pregnant and lactating women. This additional food requirement has been considered by previous study. Thus, the manuscript needs to refer to these studies in the beginning wherever it is appropriate.

Response: We do actually refer to three studies we could find that use this method/approach.

We also devote an entire sub-section (Estimates of FW and comparison with existing comparable literature) to comparing results on account of differences and similarities in the methods used by different studies.

To enumerate -

We cite Osner (1982) [#11 in references] as original energy gap study.

We cite Hall et al. (2009) [#3 in references] on which this method is based to some extent.

We cite Hic et al. (2016) [#18 in references] as a similar study, pointing out similarities and differences in methodology and associated implication for resulting numbers – including the impact on results from accounting or not accounting for children, pregnant, and lactating women.

(All relevant text from manuscript is reproduced below).

Relevant text from manuscript (3 different instances) –

"The actual method used to calculate FW is adapted from [3] and follows the energy gap approach [11]."

"Ours however is not the only study to find negative food waste values, [18] also find negative values based on the human metabolic model despite accounting for under 18 population. They interpret the negative FW values as food deficit."

"Table 3 attempts to cover the relatively recent comparable work in the field. Kcal estimates [3,18,26,27] yield more readily to such a comparison."

"While Hic et al. global FW estimate of 510Kcal/day/cap could be more precise than ours on account of including specific nutritional requirements of children, pregnant and lactating women, the claim cannot be made with certainty. This is because while taking pregnant and lactating women into account will increase energy requirements thus lowering FW levels, accounting for children (less calorie consumption than adults) will raise FW. The overall effect on relatively young countries is therefore ambiguous. At the same time it can be argued that the Hic et al. estimate is less precise than ours for a few reasons – a) their assumption regarding equal PAL values for all countries (given the very different life-styles prevalent across the world), b) inconsistent weight data across countries coming from different points in time, and c) assuming a constant unchanging BW overtime in making future projections. Assuming equal PAL for all countries likely overestimates food energy requirements for developed world thereby underestimating their FW, the opposite holds for the developing/underdeveloped nations – comparison of our FW estimates with those of Hic et al. (Table 3) lends support to this intuition."

Second, the claim that this method would be useful to monitor SDG 12 target 12.3 contradicts with the aims of SDGs. SDGs aim to reduce food loss and waste while

increasing income. However, this regression provided by this study shows that food waste increase with income.

Response: Thanks for raising this valid point. Perhaps a better framing is needed here. What we wanted to emphasize is that this method can be used to provide a benchmark for FW as it would exist if no intervention to reduce FW was undertaken. Actual FW field data can then be gathered in a country to assess how much progress has been made towards halving these numbers. Also, WRAP seems to agree with this assessment (personal communication).

Added text: "**More precisely, this method can be used to provide level of FW that would exist in absence of any intervention measures towards reducing FW in a country. This could provide a scale against which progress towards halving FW as mandated by SDG 12.3, can be assessed, to show how far a nation is from achieving the target.**"

Third, figure/table and its caption need to be standalone. Currently, this is not the case. Acronyms in figure/table are not explained in the caption.

Response: Thank you for this suggestion, we have now included full text along with abbreviation in captions, to provide clarity to any abbreviations used in the table or figure itself. **Changes are made to captions for Figures 2,3 and 4 and Tables 2,3**.

Fourth, please include an in-text citation, using the author's last name and the year of publication, when the manuscript refers to, summarize, paraphrase, or quote from another source.

Response: We understand (and are sympathetic) that it's easier to read references in a manner suggested by you. The choice of citation style however is determined as per PLoS requirements, which asks for bracketed numbers.

Specific comments

Specific Comments
Line 24-25: The studies say that one third of produced food is either lost or wasted. Since this study focuses on food waste, it would be precise to differentiate between food loss and food waste.

Response: Done. The revised text reads as follows.

"It is a widely held and cited belief that a third of all food available for human consumption is lost or wasted [1]. There is a clear distinction between food loss and food waste [1], and a part of the latter is attributable to consumers.

One problem with the estimates of food waste is that they consider only part of the picture."

Line 25-26: See also the IPCC SRCCL Chapter 5 for food loss and waste in the food system.
Response: Thanks for this reference. It has been **included** in our citations.

Line 27-28: "supply side food waste is constrained by an upper-limit as dictated by food availability" is this "supply side food waste" of food waste by consumers. It would be use single term so that readers are not confused.

Response: Fair point! Thanks, we modified text as per your suggestion.

Added text: **"We cannot waste more than the food available to eat, therefore consumer food waste is constrained by an upper-limit as dictated by food availability (FA) as determined by supply side factors."**

Line 34-38: This is unclear what "consumer specific attributes" mean. Global studies such as Hic et al 2016 and Walpole et al BMC Public Health 2012 studies food waste globally based on different country specific attributes. Please acknowledge these previous studies. Additionally, Hic et al 2016 also presents associations between food waste and Human Development Index (HDI).

Response: Okay, we added some examples of consumer attributes to clarify.

Added text: "**The demand side requires data on consumer attributes such as income, age, gender, education, residence etc**."

While the two mentioned studies add country specific information (body weight), these are not what in literature is considered in micro-data to be individual consumer attributes. On the basis of body weight alone the current study doesn't add anything new but it does when looking at consumer affluence data.

Hic et al rightly present a correlation between food surplus/deficit and HDI; by linking it to PAL, we use HDI in a manner where it actually determines food surplus or deficit.

Line 39-51: It is unclear why results are presented here in introduction instead of research questions or objective. Is this a new scientific writing style? Additionally, Target 12.3 of SDG 12 is about halving the global per capita food waste and reducing food loss. Please make this explicit.

Response: PLoS asks for a brief summary of what is achieved as part of introduction, and the concise bullet points are aimed to meet just that requirement.

Regarding using this method for SDG, changes have been made to text to include this (please see changes made in response to general comment #2).

Line 53-54: Better argument for not using FAO data would be lack of country specific values for waste. However, Hic et al 2016 provides country specific values, why the authors does not consider the use them.

Response: Our reason for not using FAO data was actually two fold. One, it uses fixed factors approach. Two, the way these fixed factors are arrived it by using two reports from the Swedish think-tank providing waste percentages for no more than 10 countries and filling the gap for rest of the world on the basis of these few countries!

Our reason for not using Hic et al are many

First, we found Hic et al very late in our analysis. Our approach was finalized by the time Hic et al was published in 2016. Yes, we have been working on it for a few years but failed to get it out given competing time demands…

Even afterwards, while we could have changed out approach, we decided not to for these reasons –

One, we agree that Hic et al does not fill the gaps to a degree that FAO does; we can not in reasonabaly criticize one source on the basis of this approach and use another following it. But even stronger argument is that we had already developed an approach which made it unnecessary to fill any gaps; if either Hic et al had come out a year earlier or we did not find a way to avoid filling gaps altogether, we would certainly use the numbers in Hic et al.

Two, using our approach lends itself to out of sample predictions and comparison for countries for which we didn’t fill the gaps in data. This and the ensuing comparison with other studies like Hall et al for US would not have been possible if we had not used our approach.

Finally, as the comparisons in Table 3 show, using this approach help us bring out comparative differences between ours and Hic et al work. As there are certainly systematic differences in the two studies (elaborated in text).

For all these reasons, we chose not to use the numbers from Hic et al. We see and appreciate Hic et al as an important piece of work and in similar vain hope to be acknowledged for adding a different approach and point of view to this literature.

Line 70-80: When this study considers constant body weight, the study would be similar to Hic et al 2016 and Walpole et al 2012. Hic et al 2016 also consider food requirement for children and additional calories requirement for pregnant and

lactating women. It is unclear how this study advances on the previous studies without considering these factors.

Response: We compare the differences in our results from accounting or not accounting for children and pregnant women extensively, but do not claim to be an improvement on this front. We do discuss what it means to not account for such demographics and what it means for counties in different stages of their demographic transition. Finally the WHO data used in this work provides national level adult body weight, which should for consistency sake, only be linked to adult food intake and not that of children.

This study however does differ, in its treatment of "constant body-weight", because the statistical sample shows enough variation in body weight, it allows for changes in bodyweight overtime for countries starting on the lower spectrum and move up the curve (figure 2) overtime as countries progress in income and weight per capita. This implicitly allows for increasing body-weights (when making predictions for a given country overtime). The underlying assumption is explicitly stated in the text.

Text from manuscript: "**Note that while overtime changes in body weight, don't explicitly feature in the regression and in obtaining a single country data, the sample variation in bodyweight across countries (Table 1) allows for this possibility**."

Line 83: It is unclear why the calculated FW data is only of sample countries is. The authors need to justify why they did not attempt to fill the data gap.

Response: we did not want to make assumptions regarding missing data, so we use only the observed data and use the variation in data to estimate a relationship which could enable us to systematically say something about countries without data.

More detailed response is provided as a response to your earlier comment regarding lines 53-54.

Line 86-87: Generally, food availability is associated with affluence or income. However, there are studies which also look at other factors. See Bodirsky et al. 2015 Plos One on Good Food Demand. Please better justify the assumption. This also holds of the relation between food consumption and affluence.

Response: Thanks for bringing this study to our notice. We see two main points that stand out from it. One, a significant time trend and two, differential impact on animal and plant-based calories. While we cannot include the first feature of the study in absence of longitudinal body weight data from WHO; we have explored the switch

from animal based towards plant based categories in richer nations and a huge uptake in animal based food in currently developing nations. This latter in our experience can very easily be reproduced by just per capita expenditure. The nature of calorie demanded or wasted (by its source – animal or plant) while interesting is beyond the scope of the current work.

Line 92-93: Need to provide some clarification of what are the sample countries. Please also make it explicit that this is a cross-sectional analysis.

Response: Information on sample countries are provided in the supplementary information. Also as suggested cross-section nature of analysis is now made explicit.

Added text: "**Absence of longitudinal data and ensuing cross-section nature of analysis, necessitates an implicit assumption underlying the out of sample and future predictions of FW - evolution of population BW and FA follows same trends in poor nations as in the rich ones**."

Line 106-109: It is unclear how the above described method can be applied to estimate waste of individual food commodity. How can consumption of individual food commodity be estimated based on energy requirement values?

Response: The commodity example is given just for illustrative purpose, as most people find it easier to think in terms of a single commodity when it comes to elasticities (owing to existing consumption literature). The equation in line can be summed over commodities to think in terms of aggregates.

We have used the framework for deriving the elasticities for individual commodities but there one needs a few additional assumptions or information if it is available. This is the topic of implementation of this new proposed concept, and is the topic matter of another work we are currently refining.

As the only point of emphasis here is to illustrate that standard notion of consumption elasticity is not quite correct, we find the current exposition here suffices to that end.

Line 123-124: It is unclear what does this "upward bias" mean.

Response: upward bias means that models using purchase elasticity as consumption elasticity over-predict consumption response as they don't account that some of the food purchased is actually wasted and not eaten. This bias really stands out when one tries to translate consumption model responses to calories, we have seen this often in our experience and have heard similar stories from other practitioners. Correcting for this bias (from our early experiments with this approach) brings the calories consumption numbers to a more believable range.

To make it more clear **an extra statement explaining the meaning of upward-bias has been added to the text** that immediately follows its appearance.

Line 131-133: Taking the average is done mainly on crop production to rule out fluctuation in food production. However, food availability is an outcome of food production, trade, stock variation, etc. Need a better justification for this argument.

Response: as you pointed out, and as stated in FAO methodology "Once estimates of the other components of the domestic supply have been made, the estimate of food available for human consumption is usually derived as a residual. Since the estimate of food available for human consumption is derived as a residual, its reliability would depend on the availability and accuracy of the other components on which it is based."

Extending the logic, taking average over food availability rules out not just the random shocks to production but also one off trade anomaly or stock offloading for example. From your comment it is not very clear to us as to what exactly is the objection to this treatment of food availability?

Line 144-148: Please make it clear that the data is available in supplementary. The supplementary files need to be referred in the main text.

Response: While we do not refer to each piece of data individually as available in supporting files. There is a statement at the end of the section indicating what you ask for – "The complete dataset and details are available as SI file S1.". In addition to general statement, following your comment **we now include the precise file name where such information can be found in the supporting material, not just in lines 144-148 but throughout the text** wherever a reference to supporting information appears.

Line 161-164: Taking into account dietary requirement of children is important because demographic characteristic also shapes a country food demand.

Response: Yes, we agree demography is one of the factors that affects food demand. There are many factors that do so. However given the dataset we work with WHO "adult" bodyweight, the objective – not projections (focus of Hic et al) but consistent estimates at a given point of time; and the implicit assumption (and not completely absurd) that countries follow similar economic growth and demographic transition, starting at different points and in different times; in our opinion is enough to justify the approach we have taken.

We do not claim it to be the perfect approach, are well aware of the implications for our estimates and we clearly outline those in our results section.

Line 166-174: It is unclear why the authors do not attempt to fill the data gap from other sources such as Demographic and Health Survey data. When the data gaps are filled, there might be differences in obtained results.

Response: We find this comment in the same direction as that regarding lines 53-54 and has been answered in detail there.

Line 185: Please refer to the supplementary material in different parts of the method section wherever it is relevant so that reader will obtain additional information from the supplementary.

Response: This comment is already addressed. Please see response to your comment for lines 144-148.

Line 217-221: This reads like table caption instead of interpretation of the results presented in the table.

Response: We fail to understand this comment. Is it the formatting that is wrong here, or did you wish for more details here? If latter, **we have provided some additional interpretation on results in Table 2**.

Table 2: GDP/Cap has not been mentioned in the method section. Please mention this also there.

Response: As GDP/Cap is not the standard data we used but only an alternative measure of consumer affluence, it should be taken as some sort of sensitivity test to show that our coefficients are pretty robust to a different measure of affluence than the one we use. As it is not part of the standard approach, it is therefore not a part of method section.

But perhaps this intention of using it as sensitivity test, needs to be made more clear, and this has now been done.

Line 231-233: See previous comment on filling data gaps.

Response: We have answered this extensively for your previous comment on data gaps.

Line 233-237: It is unclear how outliers and deviations are handled here. It is not

enough to a single value. Figure 2 clearly shows that there are countries above and below the regression lines.

Response: Outliers and deviations are dealt with in section “Data Requirements” specifically subsections on energy requirement and consumer affluence. Details on each individual case is provided in the text, regarding how we start with WHS sample listing 70 countries but we can actually only use 63 in our analysis. After accounting for cases in that section, no data points are labelled outliers.

Figure 2 only shows the calculated data pairs – FW, using equation (1) in the manuscript and the associated AIC/capita from ICP – used for the regression stated in equation (2) in manuscript. The regression curve is the regression this identified. So what you are calling deviations are actually regression residuals.

We use the curve to get FW estimates for out of sample countries and for any changes in income overtime. We do not attempt to reconcile the deviations for in-sample country in this work. But you are correct, these are important and should be accounted for in models. We did in fact device a way to add these deviations back to predictions for in-sample countries in a applied CGE model. But it is not the subject matter of this manuscript. We are happy that are asking for a lot of details, which we have and that perhaps makes for an application of this approach to a CGE model.

Line 239-241: This has been reported by previous studies, see Walpole et al 2012 and Hic et al 2016.

Response: To be very specific, Hic et al states “Food surplus is common in countries in the North, while food deficits are prevailing in the South.” It does not give a quantitative relationship between poor/richness of a country and their higher or lower food waste. But we see **your point and take it on board by changing the sentence formation to as follows – “The results are presented in Fig 3 below; with the pattern across countries confirming the general but unquantified belief that consumers in richer countries waste more food.”**

Line 336: Unclear “nutrition transition” means here.

Response: It is defined a few lines above as (quoting from text) – “nutrition transition – substituting away from calorie dense foods first to more animal products and finally to fruits and vegetables”.

Line 343-355: Using of this approach for monitoring Target 12.3 of SDG 12 would create more problem than solving it. This is because SDG aims to increase GDP but reduces food loss and waste. Currently, more food is waste with income growth. This

is one of the trade-offs in SDGs (see Pradhan et al. 2017 Earth's Future) which needs to tackle to achieve the 2030 Agenda for Sustainable Development. Thus, it would not be realistic to use the identified regression for 2001-2005 also in the future.

Response: We are not proposing achievement but measuring the extent of problem (FW) that needs to be halved.

Please also see response to your general comment #2, in this regard.

Line 356-364: What about the deviation from the fitting? Authors need to be critical about their findings by clearly providing limitation of the study.

Response: We have addressed your comment regarding deviations from fit above.

As for limitations, as they can be tied to certain features of the method proposed, they appear in the section where the approach is introduced – section on methods. We explicitly state all assumptions used in the analysis, along with any systematic bias in the resulting FW estimates obtained.

Figure 1: "Physical Activity" instead of "Activity"

Response: Figure 1 has now been **completely changed**; and also includes your suggestion.

Figure 3: Legend missing

Response: Legend in Figure 3 has now been **added**.

Figure 4: Names of Y-axes are missing

Response: Thanks, y-axis labels have now been **added**.

Reviewer 3

Reviewer #3: Review of Verma et al
This paper uses a different measure of food waste at a national scale than has featured in the literature to date. As such, it is a welcome addition to measurement of this important indicator of sustainability. The concept of affluence elasticity of food waste is a useful one in investigating this economic-behavioural relationship.

General Comments

We found your comments very helpful and asking for more. We know this work raises many curious questions on a lot of fronts, however giving too much in information on many different aspects in one manuscript can get quite confusing and run the risk of readers not understanding any of it. Therefore after much thought we decided to share only few main messages – we waste a lot more, there is a quantifiable relationship between FW and affluence, and standard consumption elasticity concept is theoretically biased. We did do quite some extra work which is not in this manuscript, we hope to expand on it in our future work.

General Comments
The paper would benefit significantly from an edit that paid careful attention to the use of English language.

Response: Thanks, we have now revised the paper keeping in mind just this point.

In Section 2 and throughout it would be useful to have further discussion of the treatment of uncertainty in the modelling and consequent results. For example, there is a discussion of the bias resulting from exclusion of children in the BW estimates (lines 161-164); how large might this, and other biases, be?

Response: We could not find readily available appropriate estimates in literature to quantify this bias in a systematic manner. The only other energy requirement (and therefore food waste) estimates at country level we could find that accounted for children and pregnant women, were in Hic et. al.

However while the estimates based on affluence levels can be compared (as done in Table 2), because the regression can predict level of food waste for level of affluence in any year; the data needed to quantify aforementioned bias is not so straight forward to compare across the two studies; for the following reasons –

1. While all our sample data on BW (used to impute FW data for regression) comes from WHO health survey, Hic et al use multitude of sources spanning years from 1986 to 2013. 27 years is a big span and cannot be consistently used for comparison with 2003 world health survey.

2. Furthermore the PALs used in calculating the energy requirements are different across the two studies, even for comparisons for a given country and year pair.
3. While they do project food waste forward, and so does the current work, they keep body weight constant in the projects while we allow for it to follow the same path as seen historically in rich countries

The calculated data on basis of equation (1) in the current work and the data from Hic et al, therefore are different for multitude of reasons apart from just former not accounting for children and pregnant women. And how much of the difference is coming from which of the contributing factors, is hard to quantify. Given this, we could only surmise about the direction of bias, which we did in comparing the estimates from the two studies.

The discussion of the results of affluence is not totally intuitive. I would have thought that elasticities would continue to increase as incomes rise, since there is less of an income constraint on meeting basic calorific needs. But perhaps there is a limit to calorific profligacy, beyond which it falls, giving an inverted-U shape. Either way, it would be worthwhile having more discussion of the phenomenon you observe. Could it be related to the Environmental Kuznets Curve concept? If so, what are the policy implications of your findings?

Response: yes, this finding that FW increases rapidly at first and slower later on, (leading to dropping elasticity) can be seen as comparable to findings that calorie intake increases rapidly with income at low levels but plateaus at higher levels. Whether or not we can compare this to environmental Kuznets curve, is not something we can say with certainty at this point. To make such an assertion, we need longitudinal data. Also, the phenomenon here needs more nuanced treatment – unlike environmental degradation, Food waste does continue to rise, in absence of any outside intervention (eg. Informational campaign); only it increases at a decelerating rate. This is a strong concept and requires more thought than the current work permits.

Policy implication: the only policy recommendations that we feel confident in making, based on our findings are –

1. While food waste is seen as a rich country problem, be aware that countries with rapid economic growth will see it emerging very soon and instead of waiting till it becomes a problem, interventions should be designed from the beginning.
2. The level of affluence around which efforts towards preventing food waste should kick-in, is around about 6.7$/day/capita (USD 2005).

These are listed in the discussion section.

Finally, has the transition to fruit and vegetables been observed anywhere? If so, it would be good to know where, and to what it can be attributed.

Response: Unfortunately there is not a vast number of studies when it comes to substantiating this claim. Calculations using data in (Drewnowski and Popkin 1997) shows that in Japan (a country where rapid growth - 1950-1970 - started ahead of rest of the world) the period between start of rapid economic growth and onset of recession (1955-1990), saw 4 of the 5 food groups showing increase in consumption. The growth in consumption using Japanese data was found to be highest for poultry, followed by meat and sugar and fats, meat and fish, fruits and vegetables. Cereals and potatoes were only items showing a decline over the same period.

A few other studies exist that are not referenced (as while the nutrition transition is an important aspect, it's not the focus of this work). (Mendoza et al. 2017) have found that in Mexico "Lower cost diets derived more calories from tortillas, tamales, beans and sugar, whereas higher cost diets contained more non-essential energy-dense processed foods and more sugar sweetened beverages, and fruits and vegetables."

Another work (Gouel and Guimbard 2019) that one of the current authors have expertise to evaluate, shows a positive though slow correlation between calories from fruits&Veg intake and income (figure and table reproduced below).

Figure 4.1 Observed and fitted (at world prices) caloric demand

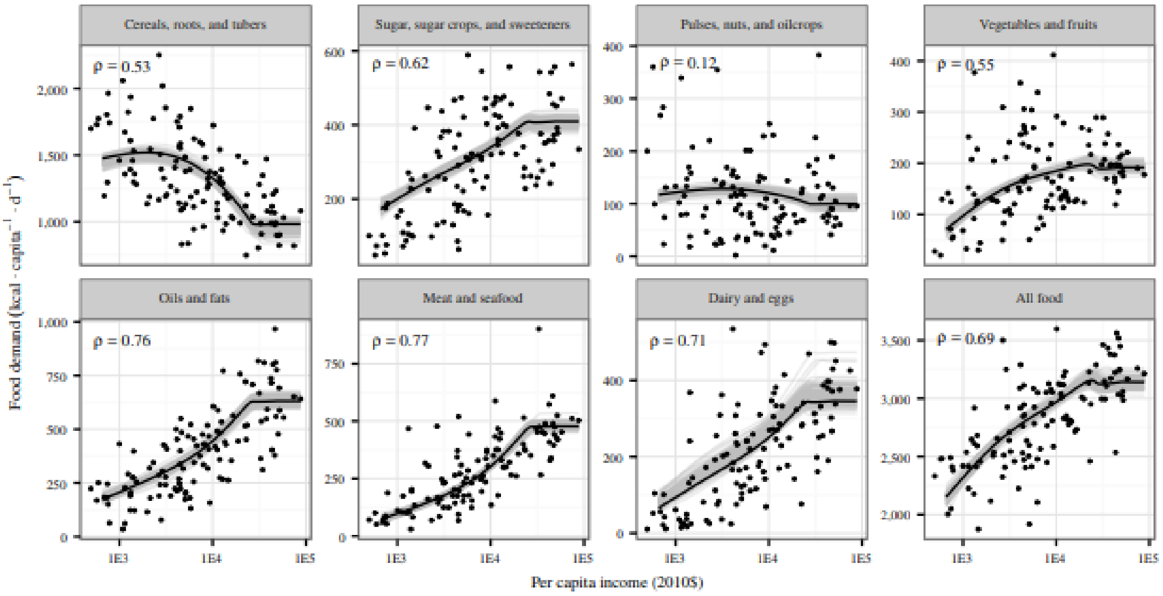

Source: FAOSTAT, WDI, and authors' calculations.
Note: ρ is the correlation coefficient of the observed values and the values fitted using individual country prices. The gray lines represent fitted caloric demand using the parameters from 100 bootstrap replicates.

Table 4.3 Decomposition of food demand change 2010 to 2050 into population and income effects (percentage change) for the trend projection

Consumption bundle	Total	Income effect	Population effect
Cereals, roots, and tubers	18.1 ± 3.2	−16.5 ± 2.3	37.5
Sugar and sweeteners	63.2 ± 11.6	24.8 ± 9.1	29.8
Pulses, nuts, and oilcrops	25.7 ± 6.4	−9.5 ± 5.1	36.1
Vegetables and fruits	53.5 ± 20.1	15.7 ± 15.2	28.3
Oils and fats	85.2 ± 10.3	44.9 ± 8.3	27.4
Meat and seafood	106.4 ± 14.2	64.3 ± 11.7	24.5
Dairy and eggs	87.3 ± 20.1	46.8 ± 16.3	25.4
Vegetal-based	35.6 ± 5.4	−0.7 ± 3.9	34.7
Animal-based	95.0 ± 14.7	54.7 ± 11.8	24.5
All food	46.0 ± 6.6	9.0 ± 4.9	32.9

Source: Authors' calculations.
Note: Central estimation and standard deviation calculated using bootstrap replicates where applicable.

The findings of (Gouel and Guimbard 2019) and calculations using data for Japan (Drewnowski and Popkin 1997) concur. We acknowledge that because higher incomes, health consciousness (following dietary guidelines) and education (knowing dietary guidelines) are highly correlated, the transition to healthier food habits (including eating more fruits and vegetables) is very likely accountable by a mix of these factors (Lallukka et al. 2007; Popkin, Siega-Riz, and Haines 2009).

Despite not being as well established as Bennet's Law (Bennett 1941) - declining importance of grains in consumption, with rising incomes), the correlation between income and fruits and vegetable intake is touched upon by some scare literature and needs more work.

Bennett, M. K. 1941. "Wheat in National Diets." *Wheat Studies of the Food Res. Inst., Stanford Univ., California* 18: 37–76.
Drewnowski, Adam, and Barry M. Popkin. 1997. "The Nutrition Transition: New Trends in the Global Diet." *Nutrition Reviews* 55 (2): 31–43. https://doi.org/10.1111/j.1753-4887.1997.tb01593.x.
Gouel, Christophe, and Houssein Guimbard. 2019. "Nutrition Transition and the Structure of Global Food Demand." *American Journal of Agricultural Economics* 101 (2): 383–403. https://doi.org/10.1093/ajae/aay030.
Lallukka, T, M Laaksonen, O Rahkonen, E Roos, and E Lahelma. 2007. "Multiple Socio-Economic Circumstances and Healthy Food Habits." *European Journal of Clinical Nutrition* 61 (6): 701–10. https://doi.org/10.1038/sj.ejcn.1602583.
Mendoza, Alfonso, Ana E. Pérez, Anju Aggarwal, and Adam Drewnowski. 2017. "Energy Density of Foods and Diets in Mexico and Their Monetary Cost by Socioeconomic Strata: Analyses of ENSANUT Data 2012." *J Epidemiol Community Health* 71 (7): 713–21. https://doi.org/10.1136/jech-2016-207781.
Popkin, Barry M., Anna Maria Siega-Riz, and Pamela S. Haines. 2009. "A Comparison of Dietary Trends among Racial and Socioeconomic Groups in the United States." *Http://Dx.Doi.Org/10.1056/NEJM199609053351006*, August. https://doi.org/10.1056/NEJM199609053351006.

Are the non-linear elasticities used in the global projection estimates. It would be helpful to make this clear.

Response: Yes, the projections are made using information on independent variable from ICP data and the coefficient estimates provided in Table 2 (The same coefficients underlie the elasticity numbers produced in Figure 4).

We have now made and explicit reference to Table 2 results in section dealing with out of sample country and global projections.

Specific comments

Specific comments
Line 16. Is the per day/capita level of expenditure just on food or on all consumer goods & services?

Response: this is total consumer expenditure on goods and services consumption of households. Per ICP definition "Actual individual consumption is measured by the total value of household final consumption expenditure, nonprofit institutions (such as NGOs and charities) serving households' final consumption expenditure, and government expenditure on individual consumption of goods and services". So it includes more than just food.

Line 73. In relation to equation 1 would it be worth stating explicitly that – as I understand it - its validity depends on the characteristics of the population remaining the same over time?

Response: You are right, equation 1 is valid for only a given point in time. However we only use data obtained from equation 1 for estimating the relationship between FW and affluence. We do not use this equation (but the relationship between affluence and FW) to make overtime projections, it is only used to calculate the initial FW data, which we had to, because we did not believe in FW numbers coming from FAO.

We make it explicit in the text that data imputed using equation 1 cannot be used to say anything about global food waste estimates - "**As our sample represents only 67% of the world population, and countries believed to waste a lot of food (e.g. US, Australia, Canada) are not present in our sample, we cannot use the population weighted average of sample FW data of 351 Kcal/cap/day (Table 1) as estimate of FW for the world in 2003. We instead use the regression results (Table 2) and world AIC value (6095 international 2005 USD) from ICP 2005 to obtain an estimate of consumer FW for the world in 2005**."

We also mention that we assume that as poor nations become rich, the evolution of Food availability and body weights follow the same pattern as observed in currently rich countries – "**Implicit assumption underlying the out of sample and future predictions of FW is that evolution of population BW and FA follows same trends in poor nations as in the rich ones**." This assumption as estimated

affluence-food waste link, enables making projections into future and out of sample countries.

Line 124. Is it possible to say anything about the broad size – in percentage terms - of the upward bias?

Response: Again, we don’t have a systematic evaluation for this. In our own calculations, we found a comparable number from a DEFRA study found calorie intake elasticity to be more than if food waste was accounted for (reference 31 in the manuscript). We do hope to use this manner of modeling consumption (accounting for food waste) to study the consumption responses overtime with economic growth in some simulation models. But currently we don’t have the bias quantified.

Page 8. The formatting is lost here. Also, the text seems to be incomplete (e.g. line 223)

Response: yes, in our submission files the formatting is okay but I see that it seems to have been lost in the file you got from PLoS. Hopefully it would be corrected! The text was left in draft and was mistakenly left as a comment to ourselves and has been removed. Thank you for pointing this out.

Lines 235-237. Is the increase in Kcal/day/capita between 2005 and 2011 as a result of increases in incomes? Or is there another explanation?

Response: In our estimates with income being the only variable in regression, all increase is attributable to it.

Lines 265-267. The argument is not quite clear to me. Are you referring to a potential double-counting estimation procedure?

Response: yes, the production losses seem to have been accounted for twice. For more details reference is provided. We have rephrased it to read as follows, hopefully providing more clarity -

“**Also Kummu et al. use food availability data which is already adjusted for country production losses, yet they account for the production losses in estimating FW [17]. This, in addition to only partial coverage of food groups, results in a downward biased estimate of consumer FW.”**

Line 308. Maybe say explicitly why the Buzby et al estimate of FW is conservative?

Response: This is claim made by Buzby et al themselves, and is well explained in Dou et al which is cited in the manuscript for interested readers. We merely reproduce this claim from this literature in the text, therefore not offering the reason.

Line 356. Does the use of the FA data from FAO lead to over-estimates? Either way, it would be good to make the direction of the bias clear.

Response: FA data from FAO affects the supply side (as oriented) of the story here. While the data has issues as detailed by many (supporting citations in text), we only wanted to emphasize the problem it poses when looking at FA data as reported for countries where subsistence agriculture is still practiced by part of the population. Given that it only accounts for production that reaches market, production for home consumption is not accounted for – this we believe is one of the causes that results in negative food waste values for some poor countries (often also the ones with agriculture partly subsistence oriented).

Therefore we can only claim that it downwardly biases (negative instead of zero or small positive value) food waste estimates for such countries; we have not made any claims on what it does for the countries where subsistence agriculture is not practiced.

Again, we only want to address the food demand side of the equation in this work; at the same time recognizing that there are issues with food supply data but not delving into those.

Lines 363-368. It would be good to have a little more on the advantages of using this new measure, in terms of its policy applications.

Response: We did not think of policy application specifically in terms of "affluence elasticity", we did however draw some conclusions on how these results can contribute towards policy actions. The one that jumped straight out is that consumer awareness should be brought to forefront in countries and geographical regions within those countries hitting a given income threshold. This kind of policy action falls in preview of preventative action before food waste actually becomes a big problem, like in the richer nations. We have changed the discussion a bit to emphasize this point. The other benefits like – being able to replicate result from a survey based study with only easily available international data; ensuring internationally comparable numbers (given the wide difference of opinions on when it comes to definition and measurement of food waste across countries); not having to fill gaps like FAO or Hic et al, are more of methodological improvements. As far as we know (personal correspondence) UN official SDG indicator for FW is currently under quantification using the same line of reasoning – linking income and food waste.

The only advantage we have seen of using the concept of affluence elasticity in our own work is more of conceptual improvement nature. When affluence elasticity of waste is accounted for in our numerical demand models, we don't get implausible results showing that calorie intake of an individual falls when they try to reduce their food waste. Accounting for affluence elasticity allows the same model to illustrate that individuals attempting to reduce waste start buying less but not reduce their physical food and calorie intake. We are still exploring what exactly it means for agricultural production, prices, land-use and emissions.
