# Supplementary material for: Consumers discard a lot more food than widely believed: Estimates of global food waste using an energy gap approach and affluence elasticity of food waste
Source: PLoS One. 2020 Feb 12;15(2):e0228369. doi: 10.1371/journal.pone.0228369 (PMC7015318; doi:10.1371/journal.pone.0228369)
Supplement: Attachment — Submitted filename: Response to Reviewers.docx [file pone.0228369.s005.docx]

Dear Editor,

Please find our responses to the reviewers below. As before we highlighted our response with grey background.

The manuscript incorporates these changes where applicable. In addition, the manuscript has been modified to address concerns regarding use of standard English. Many such changes were suggested to us by a native speaker and are reflected as tracked changes in the manuscript.

Thank you for giving us the opportunity to make revisions.

Sincerely,

Monika

(on behalf of all authors)

Enclosed

Response to Reviewer #1

Response to Reviewer #2

Response to Reviewer #3

# Reviewer #1

Reviewer #1: The authors have mostly addressed my major concerns and comments. I believe this piece can contribute to the scant but evolving literature on food waste and food loss measurement.

Response: Thank you, very much. We are glad to have been able to address all your concerns.

# Reviewer #2

Reviewer #2: General Comments

I thank the authors for their attempt to the address the comments. Although the authors provided responses to all the comments, they did not incorporate the changes in the revised manuscript. This is also visible while looking at the track changed version of the revised manuscript. Thus, I suggest considering the manuscript for publication after addressing and incorporating the following comments in the revision process.

Response: Respectfully, we disagree. We have either incorporated changes that you suggested or explained our reason for not doing so, except for the first comment below (regarding lines 53-56), which was overlooked unintentionally and has now been addressed.

First, as mentioned in the previous round, the method use for estimating food waste has been reported by previous studies which the authors do not properly acknowledge in the manuscript. Instead, the authors claim that this has not been done yet. Line 53-56 says:

“The demand side requires data on consumer attributes such as income, age, gender, education, residence etc. While there are individualistic attempts to capture the impact of consumer specific attributes [8,9] there is no study attempting this at a global level. This work is a first attempt in linking the amount of food wasted to one such consumer attribute.”

This is wrong because global studies such as Hic et al 2016 and Walpole et al 2012 cover the aspect of age, gender, and body weight. The authors need to acknowledge this.

Response: You are correct. While we do correctly point to Osner, Hall et al, and Hic et al in the main text, the sentence here does need to be changed for be accurate.

What we did in fact want to say is that the no attempt has been made to link food waste to socio-economic factors at a global scale. The individual country case studies account for socio-economic factors (income, education, location etc) but also demographic factors (age and sex). It’s in the socio-economic and not demographic context that we claim to be the first work to do so. We didn’t mean to understate the contribution of previous work you mentioned, when it comes to accounting for age and sex. The text has accordingly been rectified to read as follows –

“The demand side requires data on consumers’ socio-economic attributes such as income, education, residence, food-culture etc. There are individual attempts to capture the impact of these consumer specific attributes [8,9] using regression methods, but no studies have attempted this at a global level. While we found work using global age and sex distribution to impute global FW [10], a measure of responsiveness of FW to these demographic factors as provided by regression methods, was found missing. With the aim to fill such gaps in research, this work is a first attempt at linking the amount of food wasted to one such consumer attribute.”

We are aware of Walpole et al work however do not cite it because while they estimate global human biomass, it’s not in context of food waste; and we are not claiming to be the first to use energy balance approach globally but to derive a relationship between affluence and FW (derived using energy balance method).

Second, the study is based on the sample countries. However, the manuscript does not tell about these countries in the very beginning. As a reader, I would like to know which these sample countries are in the first place it is mentioned (see Line 99). Since readers do not know about the features of these sample countries, it is hard to justify the argument present in Line 113-114.

Response: Our decision behind this organization was to keep the method section to explain our conceptual approach and present all information on data later in the data section.

Also we don’t see any *argument* in lines 113-114, but an implicit *assumption* that body weight in poor countries follows the pattern of rich countries as they develop. It’s an assumption, just like Hic et al assumption that body weights remain constant (or everyone has Japanese or American weight). We assume that they evolve along a similar path for all countries as they grow richer. We don’t need to know the sample to make this particular assumption.

Third, I also request the authors to be critical in their projections. For example, in 2011 the global food availability is 2869 kcal/cap/day and the estimated of food waste by this study is 727 kcal/cap/day. This means 2142 kcal/cap/day is the global food consumption. This global value is below the estimated dietary requirements.

Response: We have pointed out the cons of making assumptions that we had to make in our methodology, immediately after introducing the assumption required. So we have pointed out the drawbacks that we could think of.

Regarding this particular comment, while mathematically your conclusion is correct, we do not believe it is something strictly comparable. All this number says is that ‘on average’ (disregarding distribution issues) people are consuming less than the recommended guidelines, which is not very surprising given that over 800 million people in 2010 were reported to not be able meet even their minimum dietary requirements of 1800 KCal (State of Food and Nutrition Security Report, 2019).

Mathematically (to continue your line of reasoning), Availability (2869) = consumption + waste

You claim that Consumption (C) should meet dietary guidelines(DG), and definitely Minimum requirements (MR).

Let’s say there are 6 groups of people, very rich (over consuming), so C>DG. Very poor with C<MR (the 822 million). and 4 groups in between with let’s say just above or below the DG. Now whether or not the average is dominated by over consumption of richer group or gross under-consumption of the poor, depends on population distribution.

If then the share of population in poor countries not meeting the DG is bigger, why is it very surprising, even with over consumption in smaller richer population section, that the global average consumption (not availability) is below the DG?

Also, looking into this sort of comparison would require, not just the numbers and share of global population malnourished and overfed but also looking into extent (depth) of their despair/indulgence – an interesting question but not the current subject matter. So we don’t see how these numbers contradict any widely believed facts - such as reducing waste would ensure enough to meet DG for everyone.

Fourth, the recent report from FAO, The State of Food and Agriculture (SOFA), focuses on food loss and waste. Thus, it would be relevant to discuss the finding of this study also in the context and finding from SOFA.

Response: Please see our response to specific comment regarding SOFA, below.

Specific Comments

Line 115: What does “Genetic differences” mean? Please explain this to reader.

Response: We have now provided a citation for interested readers.

Line 232-233: Since the study used the average value between 2000 and 2005, it would be better to say “circa (ca.) 2003 instead of 2003.

Response: The values between 2001 and 2005 were used ***only for averaging out any production and export shocks/anomalies*** in the FAO food availability data. The WHS data on bodyweights refers to a single year (2003) in which the survey was actually conducted. Therefore we chose keep it at 2003.

Line 247: GDP/Cap has not been mentioned in the method section. Please mention this also there. The authors’ repose was “But perhaps this intention of using it as sensitivity test, needs to be made more clear, and this has now been done”. However, when this is used as sensitivity test, this needs to mention in method. There is no sentence on “sensitivity test” in the method section.

Response: No, we did not mention it in the methods section, for reasons^[1]^ we explained in responding to first round of your comments.

However to accommodate your concerns this sentence (in **bold**) was included in the results section (where GDP is first mentioned) during first revisions – “Using alternative variables as a measure of affluence – ln(GDP/cap) and ln(AIC/cap) from ICP – gives similar results but AIC shows a slightly better fit (Table 2, column 5). **This shows that our coefficient estimates are robust to the choice of measure of consumer affluence.**”; explaining as to why we were suddenly also providing GDP/cap as an alternative measure of affluence. We did not include the word sensitivity but robust.

Line 269-270: When possible it would be better to provide range or standard deviation for the estimates

Response: We have provided a range on the coefficient estimates (table 2). But we decided to keep a single number as FW estimate for two reasons –

a) we are already having to give two numbers because our comparison group (Kummu, Hic, Hall, Buzby) ranges in terms of years for which they provide estimates; and
b) our comparison group comes without a within scenario range (and we do not have scenarios). Comparison of range from scenarios with range on estimates would not be comparing apples to apples.

Furthermore providing a range in addition to 2 years (2005 and 2011) would add to more confusion than clarity. From Table 2 of course, it is clear there that there is a standard error and therefore an associated range.

However we see your point and have now made a clarifying addition to text – “Note that these estimates of FW are derived using the central estimates of the regression coefficients in Table 2, and a range around these cold be constructed using information in the said table.”

Line 272-274: This is no surprise and the study use the relation of increase food waste with increase income. However, the relation is not able to capture rich countries wasting less food, e.g., Japan.

Response: One, Japan was not a country in our sample. Two, no regression can ever (unless calibrated) predict all cases and in particular special cases. In case of Japan, it is a country with a specific food culture where small portions and plates, single dishes instead of buffets, and eating out, is very prevalent and unless a regression includes food culture as an explanatory variable it would not be able to reproduce numbers for Japan. The emphasis of this work is to capture monetary dynamics, *cetris paribus*, in absence of any other factors that contribute towards reducing food waste. This analysis wasn’t made to and will not capture any reductions that come from factors such as food culture, consumer awareness etc. It won’t do so for rich or for that matter even poor countries.

And while we did not name Japan specifically we did mention caution such cases in the discussion (original text, not tracked change) “Using easily available standard macro-economic data like expenditure, does not undermine the importance of detailed country specific micro-data analysis, to understand the influence of specific factors like education, consumer awareness, attitudes, cultural food practices and preferences etc.; only that detailed internationally consistent data on such factors is harder to gather.”

Line 374: Although in the response, the authors mentioned: “It is defined a few lines above as (quoting from text) – “nutrition transition– substituting away from calorie dense foods first to more animal products and finally to fruits and vegetables”.”. This change is not visible in the manuscript.

Response: The change is not visible as a tracked change because the definition (i.e. text in quotes “” reproduced by you above) was already present in the original manuscript. Hence it didn’t require any change. We merely quoted a section from original text that you had perhaps overlooked.

Line 383-385: Please highlight that this is the case under the current trend.

Response: Just to make sure we understand what you are referring to, lines 383-385 are – “Our approach to obtaining quantitative estimates of link between FW and consumer affluence, provides a way of obtaining comparable FW estimates for countries without detailed surveys, based only on easily available consumer expenditure or GDP data.”

We don’t understand this particular comment. This text explains what this approach does. Is it current trend in research to estimate global and national FW using GDP data?

Line 392-394: Regarding food waste index, please also see SOFA 2019.

Response: We are aware of SOFA, and have been consulted by FAO for the report. From both SOFA and our communication with UNEP, we know that SOFA only provided an index for Food loss and not for food waste. So we cannot compare our results with SOFA because SOFA didn’t provide any!

Line 395-397: The authors may include that “SDG 12 (Responsible Consumption and Production) could be the bottleneck for sustainable development (Pradhan et al. 2017)”

Response: Thank you for drawing our attention to Pradhan et al 2017 (in both rounds of your comments), but the subject matter here is not *tradeoffs*; we would be very happy to include this excellent citation when we look into the tradeoffs.

# Reviewer #3

Reviewer #3: Thank you for your responses to my comments. They are reassuring. Your response to my query about the transition to fruit and vegetables is useful; it would be good to include a condensed version of this in the text. I also think a revision by a native English speaker is still required.

Response: Thanks, we can certainly include a brief summary with references. The text below appears in the manuscript now, to give a little more information to readers interested in knowing about this transition:

“Although we have not explicitly accounted for nutrition transition and it is beyond the scope of this work, as higher incomes, health consciousness (following dietary guidelines) and education (knowing dietary guidelines) are highly correlated, the transition to healthier food habits (including eating more fruits and vegetables) is very likely accountable by a mix of these factors [32,33]. And despite not being as well established as Bennet’s Law (declining importance of grains in consumption, with rising incomes) [34], the correlation between income and fruits and vegetable intake is touched upon by some scare literature [31,35,36] and needs more work.”

On the second issue, we managed to coax a British colleague to proofread the manuscript and make changes. These changes appear throughout the manuscript as tracked changes and it hopefully reads better.

1. Text reproduced from answers to first round of comments – “As GDP/Cap is not the standard data we used but only an alternative measure of consumer affluence, it should be taken as some sort of sensitivity test to show that our coefficients are pretty robust to a different measure of affluence than the one we use. As it is not part of the standard approach, it is therefore not a part of method section.”
